# Bacteriological Profile of Urine in Patients with Different Types of Kidney Stones in a Tertiary Care Hospital: A Descriptive Cross-sectional Study

**DOI:** 10.31729/jnma.5226

**Published:** 2020-11-30

**Authors:** Srijana Ranjit, Amit Kumar Singh

**Affiliations:** 1 Department of Microbiology, Kathmandu University School of Medical Sciences Dhulikhel Hospital, Dhulikhel, Kavre, Nepal; 2 Department of General Surgery, Kathmandu University School of Medical Sciences Dhulikhel Hospital, Dhulikhel, Kavre, Nepal

**Keywords:** *bacteriology*, *kidney stones*, *urinary tract infections*

## Abstract

**Introduction:**

The association of bacteriology in the pathogenesis of urolithiasis is a known and fact. The urinary tract stones being the most common problem that brings the patient to the surgical outpatient department; it is important to know the relation between the types of stone and the organism isolated from the urine for better management of the patient. The aim of this study was to find out the urine bacteriological profile of patients with kidney stones.

**Methods:**

This is a descriptive cross-sectional study done over 18 months in a tertiary care hospital in Nepal. Ethical clearance was taken from the Institutional Review Committee (No: 03/16). Preoperative urine cultures were done routinely in all the patients who agreed to take participate in the study. The biochemical stone analysis was done. Urinary microbial floras and stone composition were noted. Data entry and analysis was done using Statistical Package for the Social Sciences version 25.0.

**Results:**

Among 107 patients, kidney stones were more common in males and most of the patients were in their 2^nd^ to 4^th^ decade. Female patients 45 (42.05%) had more predilections towards the urinary tract infection. Among 15 (14.01%) positive cultures, *Escherichia coli* 10 (67%) was the most common organism isolated followed by *Klebsiella;* 4 (27%), and *Pseudomonas;* 1 (6%).

**Conclusions:**

Thus, we would like to state that *Escherichia coli*, though being a non-urease producing organism, is a major organism isolated in the preoperative culture of urine in a patient with kidney stones.

## INTRODUCTION

Uroliths are known almost from the beginning of human civilization.^1^ Urolithiasis is one of the most common problems that a patient visits the urology outpatient department of our hospital. Its prevalence ranges from 7% to 13% in North America, 5%-9% in Europe, and 1 %-5% in Asia.^2,3^ Geography, climate, diet, fluid intake, genetics, gender, occupation, and age are the variables that significantly affect the prevalence rate.

Urinary tract infection is the most common and important risk factor for urolithiasis.^3,4,5^ As high as 28% of all Urinary stone disease has been associated with urinary tract infection (UTI).^6^ However, there is controversy regarding the role of urea splitting organisms in the formation of renal stone.^7^

Understanding the bacteriology of stone formers can allow improved patient care and possibly prevention of recurrence and formation of new stones.^4^ Thus this study aimed to find out the urine bacteriological profile of patients with kidney stones.

## METHODS

This is a descriptive cross-sectional study, done for 18 months in a tertiary hospital in Nepal. After getting ethical clearance from the ethical team (No: 03/16); All the patients who were planned for surgical removal of kidney stones were enrolled in the study. Written consent was taken and a preoperative bacteriological profile of mid-stream urine was done. Every patient who gave consent underwent postoperative biochemical analysis of the stone. Those patients who were not fit for surgery and those who didn't want to do a biochemical analysis of the renal stone were excluded from the study. The convenient sampling method was used to calculate the sample size with a prevalence of 50%, as:

$$\begin{array}{ll} \text{n} & \text{= }{\text{Z}}^{\text{2}}\times \text{p}\times \text{(1}-\text{0.5)}/{\text{e}}^{\text{2}}\\ & \text{= }{\left(1.96\right)}^{\text{2}}\times 0.5\times 0.5/{(\text{0.1)}}^{\text{2}}\\ & \text{= }96 \end{array}$$

Where,

Z = 1.96 at 95% confidence interval
p = population proportion, 50%
q = 1-p
e = margin of error, 10%

Taking a 10% non-respondent rate, the final sample size was 105.^6^ Thus, our study included 107 patients during the study period. These all stones were sent for biochemical analysis using a standard protocol.^8^ All the patients enrolled in the study were asked to fill the proforma; containing general information about them, urinary symptoms, and signs.

Data were collected via paper-based questionnaires and the data were entered and analyzed using the Statistical Package for the Social Sciences (SPSS) version 25.0 for Windows. Demographic variables were analyzed using descriptive statistics. Data were collected throughout the study period to meet the sample size for the study.

## RESULTS

In the study out of 107 patients; 73 patients had urinary tract infection. Out of these 73 patients; 15 (20.05%) were culture positive. Out of 107 patients, 62 (57.95%) were male and 45 (42.05%) were female. The mean age of the participant was 38.94 ± 14.01 years (Table 1).

**Table 1:** Socio-Demographic details of the participants (n=107).

Variables		Frequency n (%)
Gender	Male	62 (57.95)
	Female	45 (42.05)
Age	<20 Years	7 (6.54)
	20-40 Years	58 (54.20)
	40-60 Years	36 (33.60)
	>60 Years	6 (5.60)
	Morbidly Obese	1 (0.8)
Occupation	Farming (Agriculture)	35 (32.7)
	Business	22 (20.5)
	Household	21 (19.6)
	Officer	18 (16.82)
	Labor	2 (1.86)
	Others	9 (8.41)

In the study group, 68 (63.55%) patients were found to have clinical/laboratory UTI (Pus Cells in Urine: more than or equal to 5/hpf) (Table 2).

**Table 2:** Clinical and laboratory evaluation of the patients.

Variables	Frequency n (%)
Symptoms	
Urinary tract infection (UTI)	73 (68.22)
Culture positive UTI	15 (14.01)
Presenting complaints	
Pain	100 (93.45)
Gross hematuria	9 (8.40)
Burning micturition	45 (42.0)
Frequency	43 (40.1)
Nausea	23 (21.4)
Vomiting	15 (14.0)
Fever	10 (9.0)

**Figure 1:**
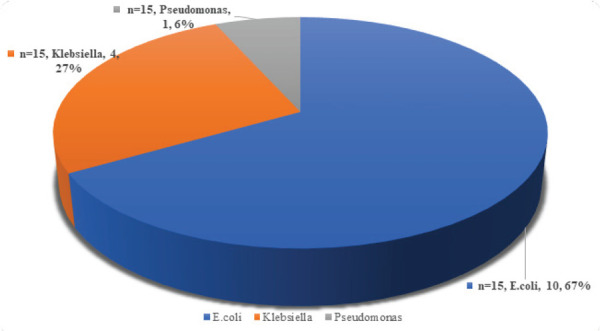
Culture positive cases.

The total number of culture-positive among 107 patients were 15 (14.01%). Among them, E. Coli was seen in 10 (67%) cases, Klebsiella in 4 (27%), and Pseudomonas 1 (6%) (Figure 1).

**Figure 2:**
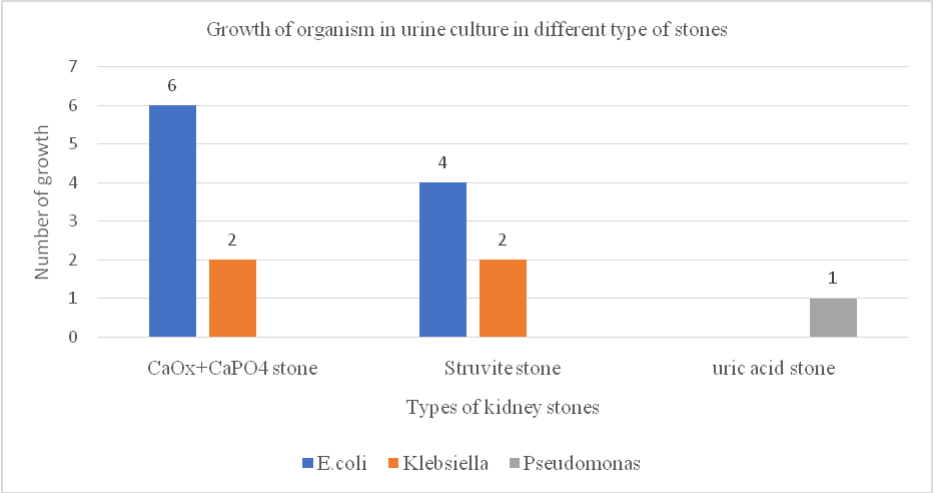
Microorganism in different types of stones.

We also found that 23 (21.49%) patients had a history of recurrent UTI in the last two months and out of which 15 (14.01%) patients were having UTI at the time of study as well (Figure 2).

**Figure 3:**
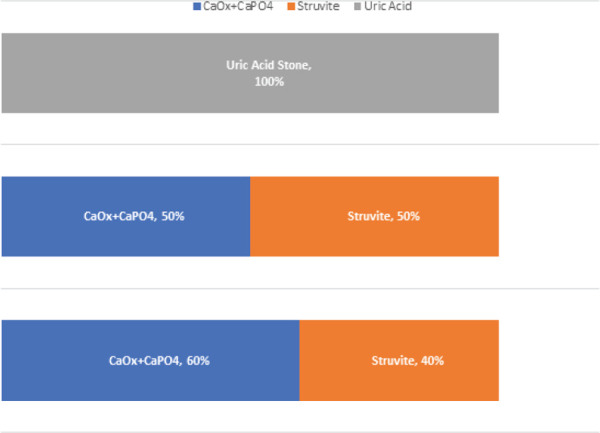
Urine bacteriology of different types of stones.

In biochemical analysis; the most common type of stone was a mixed type of stone formed by calcium oxalate (CaOx) and calcium phosphate (CaP04) as seen in 80 (74.77%), followed by uric acid stone in 13 (12.2%), struvite in 12 (11.22%), and cysteine 2 (1.87%). Interestingly, 8 (53.33%) of preoperative Urine culture-positive patients had mixed CaOx + CaPO4 stone, 6 (40.00)% of culture-positive had Struvite stone and 1 (6.67)% had Uric Acid stone (Figure 3).

Calcium oxalate + calcium phosphate has 8 Culture positive organism in urine; out of which 6 culture had E.coli and 2 culture had Klebsiella. Struvite stone had 6 culture positive organism (4 cultures with E.coli and 2 cultures with Klebsiella) and uric acid stone had one organism that is Pseudomonas grown in pre-urine culture. Figure 2 shows the different microorganism grown in different types of stones.

## DISCUSSION

The study has been conducted on 107 patients with kidney stones to find the bacteriological profile of preoperative urine culture and types of stones. In this study majority of the patients were male with male to female ratio being 1.3:1. Our study is similar to the study done in the past by various authors suggesting male predominance in stone formers. Seitz C et al.^9^ suggested the prevalence and incidence of kidney stones are increasing and there is a male predominance. However, one study done in Nepal suggested female stone former are more in Nepal.^10^ Out of 73 UTI patients, 43 were female and 30 were male. UTI in females occurs more often than males at the ratio of 8:1.^11^ Approximately 50-60% of the female population report UTI once in their lifetime.^2,11^ Frequent bacterial contaminations along the short urethral canal from the perineum and the change in genitourinary tract mucosa after menopause may cause colonization by coliform organisms which is perhaps the cause of frequent UTI in females.

In our study we found preoperative urine culture to be positive in 20.54% of patients with clinical or laboratory UTI; E. coli is the most common bacteria (67.00%) isolated, followed by Klebsiella (27.00%) and Pseudomonas (6.00%). A study was done in Nepal also found E. coli to be the commonest organism isolated in the urine culture.^12^ Many other studies also have reported that E. coli being the most common bacteria isolated from the urine culture of patients.^13,14^

The mixed stone containing calcium oxalate and calcium phosphate contained E. coli as a common organism (75%) grown in culture and the rest 25% was Klebsiella. In struvite stone, four patients with culture-positive showed E. coli and two patients showed Klebsiella. In uric acid stone, there was one culture-positive which was Pseudomonas. Many studies correlated urine culture and stone Culture with types of stone. In our study, we didn't culture the stone, but the urine culture is similar to other studies with E. coli as the commonest pathogen.^11,15^ Literature suggests that E. coli is not urease producing organism thus least likely to cause renal stone.^15^ However, in our study E. coli is the commonest organism isolated in the urine culture of stone formers. This is supported by other studies as well.^11^ Al-dabbagh AA found E. coli in 40% of preoperative positive urine cultures.^16^ The recovery of E. coli from the majority of positive urine culture of stone formers suggest that even the non-urease producing organism has something to do with the stone formation.^5,17,18^

There are studies in the literature that suggest that organisms isolated from Urine culture are different from the stone culture and they advise for stone culture too.^5,8,11^ Further study might be warranted to know this correlation. The large sample size and analyzing the stone with advanced methods like spectrometry might be more scientific if the facilities provided and cost-effective.

## CONCLUSIONS

Our study shows that urolithiasis is more common in males and most of the patients are in their 2^nd^ to 4^th^ Decade. Female patients had more predilections towards the urinary tract infection. E. coli is the most common organism isolated from the preoperative urine culture followed by Klebsiella and Pseudomonas. Mixed stone composed of CaOx and CaPO4 is the most common type of kidney stones followed by struvite stone and uric acid stone. Culture positive urine is more with mixed stone (CaOx and CaPO4) and the major organism isolated with this type of stone were E. coli. Thus, we would like to state that E. coli though being non-urease producing organisms; are major organisms isolated in the preoperative culture of urine.
